# Phytochemicals and Cancer Treatment: Cell-Derived and Biomimetic Vesicles as Promising Carriers

**DOI:** 10.3390/pharmaceutics15051445

**Published:** 2023-05-09

**Authors:** Sara Baldassari, Alice Balboni, Giuliana Drava, Daniela Donghia, Paolo Canepa, Giorgia Ailuno, Gabriele Caviglioli

**Affiliations:** 1 Department of Pharmacy, University of Genova, 16148 Genova, Italy; sara.baldassari@unige.it (S.B.); alice.balboni@edu.unige.it (A.B.); giuliana.drava@unige.it (G.D.); daniela.donghia@edu.unige.it (D.D.); 2 Department of Physics, University of Genova, 16146 Genova, Italy; paolocanepa@unige.it

**Keywords:** cancer, plant derivatives, extracellular vesicles, cell-derived vesicles, hybrid vesicles, biomimetic nanoparticles

## Abstract

The majority of anticancer agents currently used derive from natural sources: plants, frequently the ones employed in traditional medicines, are an abundant source of mono- and diterpenes, polyphenols, and alkaloids that exert antitumor activity through diverse mechanisms. Unfortunately, many of these molecules are affected by poor pharmacokinetics and limited specificity, shortcomings that may be overcome by incorporating them into nanovehicles. Cell-derived nanovesicles have recently risen to prominence, due to their biocompatibility, low immunogenicity and, above all, targeting properties. However, due to difficult scalability, the industrial production of biologically-derived vesicles and consequent application in clinics is difficult. As an efficient alternative, bioinspired vesicles deriving from the hybridization of cell-derived and artificial membranes have been conceived, revealing high flexibility and appropriate drug delivery ability. In this review, the most recent advances in the application of these vesicles to the targeted delivery of anticancer actives obtained from plants are presented, with specific focus on vehicle manufacture and characterization, and effectiveness evaluation performed through in vitro and in vivo assays. The emerging overall outlook appears promising in terms of efficient drug loading and selective targeting of tumor cells, suggesting further engrossing developments in the future.

## 1. Introduction

According to the World Health Organization (WHO), nearly one in six deaths worldwide are due to cancer. Cancer is a large group of diseases characterized by the uncontrolled proliferation of the so-called cancer stem cells, capable of generating a malignant cell population that proliferates with no control, potentially expanding to a whole organ or, in the worst cases, to other tissues, forming metastases [1,2]. Among the numerous possible causes behind this anomalous cell behavior, the WHO has identified random somatic mutations, reactive oxygen species (ROS), ionizing radiation, and chemical and biological agents as the most common triggers of malignant transformation of cells [3]. One of the first aspects concerning the presence of cancer is its detection. Besides the most common identification methods, the development of rapid and non-invasive techniques for detecting cancer remains a sought-after goal; for example, in recent years the development of single or multiple techniques [4,5,6,7] exploiting biosensors for the detection of cancer biomarkers is gaining increasing interest.

Unfortunately, in spite of progress in the knowledge of molecular and biological mechanisms, many cancers are still characterized by very poor prognosis [8], as these advancements have not been translated (with a few exceptions) into effective diagnostic and therapeutic treatments. The various therapeutic approaches, which may involve surgery, immunotherapy, radiotherapy, chemotherapy, photodynamic therapy, or a combination of these [9], suffer from limited bioavailability, fast clearance, rapid emergence of drug resistance, and side effects due to lack of specificity. Different strategies have been investigated to increase the potency of the conventional anticancer drugs and limit the toxicity towards healthy cells, such as the development of inactive prodrugs or the incorporation of the actives into delivery systems, also opening the way to actively targeted therapies [10].

On the other hand, the impelling need for novel and more effective compounds for cancer treatment has driven research towards alternative sources, especially plants [11]: nowadays about 60% of the commonly used anticancer drugs were obtained from natural sources [12,13], either inspired by traditional medicine [14] or revealed by experimental findings [15]. Herbal medicines in the form of teas, tinctures, powders and poultices have been used for over 5000 years [10] in traditional medicines, still diffused in 88% of all countries, according to the WHO Global Centre for Traditional Medicine.

Medicinal plants are rich in diverse phytochemicals, such as flavonoids, terpenoids, alkaloids, saponins, lignins, coumarines, stilbenes, and taxanes [9,16], which are present in different parts of the plant, such as flowers, flower stigmas, pericarp, fruits, seeds, sprouts, roots, leaves, embryo, bark, and rhizomes [9]. Some of these compounds may exert anticancer effects through different mechanisms, for example through the inhibition of cancer-cell activating pathways, potentiation of DNA repair mechanisms, induction of antioxidant action, and stimulation of protective enzyme formation [9,17]. To identify such active compounds, a multistep process involving different fields of expertise is required [14,18].

Despite their beneficial potential, the use of phytochemicals for cancer treatment is frequently hampered by bioavailability issues, due to poor water solubility, high metabolization rate and low chemical stability [19,20,21,22,23], issues that may be overcome by including the phytochemical in a nanoformulation [24,25,26,27,28,29,30,31]. Among the most investigated nanovehicles are liposomes and lipid-based nanosystems [32,33,34,35,36,37], micelles [38,39,40,41,42], polymeric nanoparticles (NPs) [43,44,45], niosomes [22,46,47,48] and nanosponges [49,50,51,52,53,54]. Moreover, besides NPs, nanosizing techniques leading to the formation of active nanocrystals have been employed to formulate camptothecin [55,56], curcumin [57], quercetin [58], and paclitaxel [59,60].

Recently, cell-derived vesicles have attracted a great deal of interest in the field of drug delivery. Extracellular vesicles (EVs) are a heterogeneous class of membrane-derived vesicles secreted by all cell types [61] which, by vehiculating proteins and genetic material, are involved in cell-to-cell communication [62]. Nevertheless, controversial data have been reported about the role of cancer cell-secreted EVs, either promoting tumor progression [63], or exerting an antitumor effect [64].

**Figure 1:**
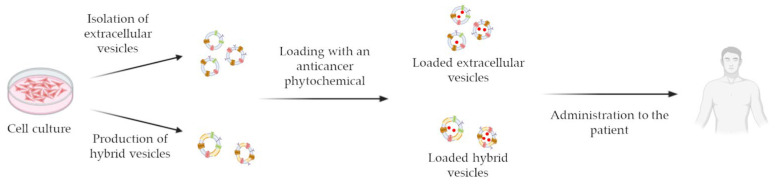
Scheme representing the production and active loading of EVs or bioinspired NPs, for the treatment of cancer-affected patients.

EVs, and in particular exosomes (EXOs), i.e., 50–130 nm EVs originating from the exocytosis of multivesicular bodies [65,66], are currently being investigated as drug delivery tools (Figure 1), since they are nano-sized, biocompatible, non-toxic, scarcely immunogenic, and endowed with targeting and homing abilities [62]. However, besides the controversial evidence mentioned above, several technical issues are hampering the clinical translation of EVs and EXOs into drug carriers, i.e., the considerable structural heterogeneity and complexity of these vesicles, the low production yield, the difficulties in drug loading and in developing standard and scalable GMP procedures for vesicle isolation and purification [62].

**Figure 2:**
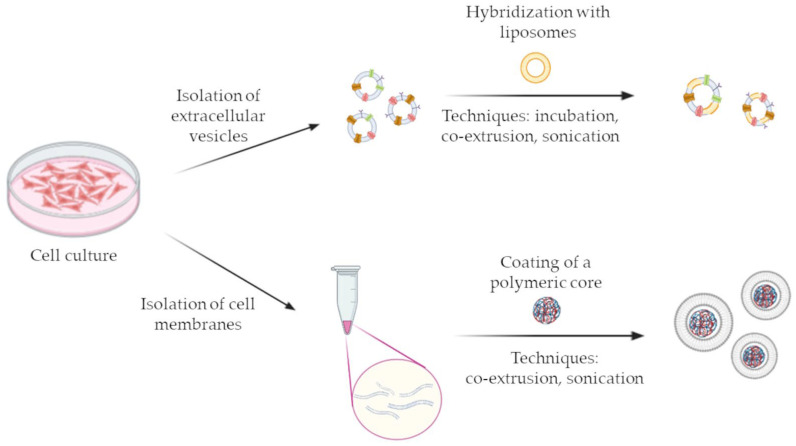
Formulation techniques for the production of bioinspired EV-like NPs.

An intriguing alternative to natural vesicles is represented by bioinspired EV-like NPs, for example EXOs obtained from a serial extrusion process of a parent cell membrane suspension through decreasing pore size membranes or from hybridization of EXOs and liposome membranes [67,68,69]. Examples of the possible formulation techniques leading to the production of bioinspired EV-like NPs are depicted in Figure 2. The combination of these innovative cell-derived nanotechnologies with the anticancer efficacy of plant-derived actives might lead to the development of novel powerful therapeutic tools, characterized by selectivity, low immunogenicity and reduced side effects.

On the wave of the growing interest in these promising drug delivery systems, this review will focus on the technological aspects of the recently developed cell-derived nanosystems for the delivery of phytochemicals endowed with anticancer activity, classified on the basis of the plant-derived active vehiculated by the nanoformulation, but also differentiated on the basis of the type of carrier, its preparation and loading procedure and the functionalization, if present. The consulted papers are listed in Table 1.

## 2. Plant Derivatives Vehiculated in EXOs and Biomimetic Hybrid Vesicles

### 2.1. Paclitaxel

Taxanes are a class of diterpenes originally isolated from plants of the yew family (Taxaceae). The yew trees began to attract chemists’ attention in the mid-1800s, when a powder named taxine was isolated, but it was only in the early 1960s that the first structures of taxane diterpenes and pseudoalkaloid diterpenoids were elucidated [103]. Starting from the 1990s, three taxanes have been employed in the treatment of various cancers: paclitaxel (PTX), which was approved for medical use as Taxol^®^ in 1993; docetaxel, a synthetic derivative of PTX [104] that entered the market in 1995 under the brand name Taxotere^®^; and the semi-synthetic cabazitaxel [103], marketed since 2010 under the trade name Jevtana^®^, which was demonstrated to be superior to PTX and docetaxel, due to the reduced affinity for the multidrug-resistant P-glycoprotein (P-gp) [105]. Taxanes exert anticancer activity by binding β-tubulin and promoting the formation of irreversible assemblies of microtubules, slowing or blocking mitosis and inducing apoptotic cell death [106]. Moreover, taxanes alter multiple cellular oncogenic processes, including angiogenesis, inflammatory response, and ROS production [104].

The clinical use of taxanes is hampered by some serious side effects, including myelosuppression, neuropathy, allergic reactions, and gastrointestinal toxicity, but also by the emergence of drug resistance that occurs according to different mechanisms, such as overexpression of efflux transporters, defective apoptotic machineries, alterations of drug targets, and barriers in drug transport [107]. Formulating taxanes in nanoparticulate systems, and in particular bioinspired-nanosystems, might help in overcoming these issues.

Several groups focused their research on taxane-vehiculating EXOs, attempting to employ vesicles derived from different sources and either loading PTX in the parent cells or incorporating it into isolated vesicles by incubation, possibly aided by sonication. For example, Pascucci et al. [70] developed PTX-loaded EXOs starting from murine mesenchymal stem cells (MSCs) derived from bone marrow, selected for their easy isolation and in vitro expansion procedures and for their ability to migrate into the tumor mass after systemic injection [108,109]. Indeed, the authors of this paper, after observing that PTX-primed MSCs exerted antitumoral effects on different types of tumor cells in vitro and in vivo [110], wondered whether PTX release from the cells was mediated by EVs. For the priming, MSCs were exposed to PTX for 24 h, and then the EVs were isolated from the cell medium by differential centrifugations. Transmission electron microscopy (TEM) observation of the isolated EVs revealed a vesicle population with size ranging from 10 to 150 nm, suggesting that the isolation procedure led to the selection of a subpopulation mainly composed of EXOs, and the presence of PTX in the EVs was assessed by HPLC analysis and Fourier-transform infrared (FTIR) spectroscopy. The isolated EVs exhibited an antitumor effect on human pancreatic adenocarcinoma cells by inducing up to 80% of tumor growth inhibition vs. controls.

In addition, Melzer et al. [71] selected MSCs (of human origin, in this case) as a source of EXOs, and primed them with PTX. In the nanoparticle tracking analysis (NTA), the isolated PTX-EXOs exhibited a negative zeta potential (−43 mV) and an average diameter of 204 nm, slightly larger than the one of the empty EXOs obtained from non-primed MSCs, while the presence of protein biomarkers on the vesicle surface was assessed using immunoblot analysis; moreover, the PTX-primed cells secreted higher amounts of EXOs, compared to non-primed cells. The concentration-dependent cytotoxicity of the formulation was evidenced by a fluoroscan assay performed on three human cancer cell lines (A549 lung cancer, SK-OV-3 ovarian cancer, and hybrid MDA-hyb1 breast cancer cells), while the administration of PTX-EXOs to breast tumor-bearing mice resulted in a 64% reduction in the tumor weight, measured after animal sacrifice.

**Figure 3:**
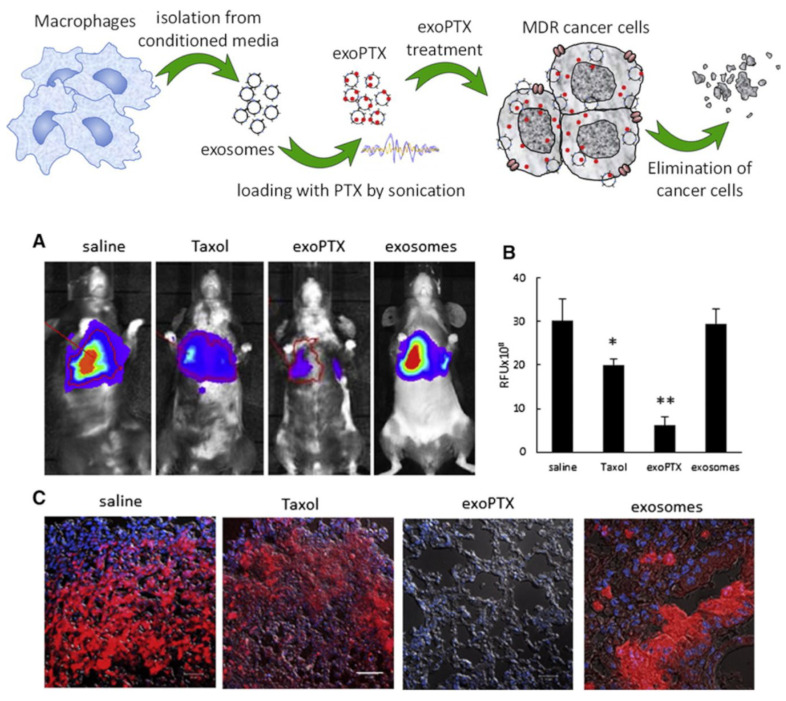
Schematic representation of PTX-EXOs preparation. Inhibition of metastases growth in mouse lungs upon PTX-EXOs treatment. C57BL/6 mice were IV injected with 8FlmC-FLuc-3LL-M27 (red) cells to establish pulmonary metastases; 48 h later, the mice were treated with PTX-EXOs, or taxol, or saline, or empty sonicated exosomes as a control, and the treatment was repeated every other day, seven times in total. Representative IVIS images were taken on day 21 (**A**). Statistical significance of metastases levels from IVIS images in lungs of treated animals compared to control mice is shown by asterisk (* *p* < 0.05; ** *p* < 0.005) (**B**). At the endpoint, 21 days later, mice were sacrificed, perfused, and lung slides were examined using confocal microscopy (**C**). Bar: 10 μm. Reproduced with permission from [72].

With the aim of circumventing the multiple drug resistance limiting the efficacy of many chemotherapeutics, Kim and his group [72] loaded PTX into murine macrophage-derived EXOs. After isolating the vesicles using a commercial kit, PTX loading was achieved either by incubation, electroporation, or sonication. EXO-PTX obtained by sonication, examined by dynamic light scattering (DLS), exhibited higher mean hydrodynamic diameter (288 nm) and lower zeta potential (−14 mV), compared to the ones obtained by incubation and electroporation; the sonication procedure also led to the highest drug loading (DL = 28%, measured by HPLC), while not modifying the protein content, as confirmed by Western blot analysis. Atomic force microscopy (AFM) showed that the EXOs retained their round-shape morphology after PTX loading through the different procedures, and dialysis followed by HPLC analysis evidenced a three-hour burst release of PTX, followed by a sustained release. An evident accumulation of 1,1′-dioctadecyl-3,3,3′,3′-tetramethylindo-carbocyanine perchlorate (DiI)-labeled PTX-EXOs in a murine Lewis lung carcinoma cell subline (3LL-M27) was assessed by confocal imaging. Moreover, an MTT (3-(4,5-dimethyl-2-thiazolyl)-2,5-diphenyl-2-H-tetrazoliumbromide) assay evidenced that the PTX-EXOs exerted higher antitumor effect on resistant MDCK canine cancer cells expressing high levels of P-gp, compared to PTX alone. Ex vivo confocal images revealed that 98% of EXOs co-localized with the lung metastases which were excised from mice intranasally administered with PTX-EXOs, indicating the efficient in vivo targeting of the nanosystem; this accumulation also resulted in the reduced progression of pulmonary metastasis in treated mice, monitored through bioluminescence of the lung cancer cells used to create the cancer animal model (Figure 3).

In a following work [73], the authors optimized the PTX-EXO formulation by incorporating aminoethylanisamide-polyethylene glycol (AA-PEG) in the vesicle membranes, providing stealth properties [111] and the ability to target the sigma receptor overexpressed in lung cancer cells [112]. To prepare the targeted PTX-loaded EXOs (AA-PEG-EXO-PTX), AA-PEG was added to a mixture of EXOs and PTX, followed by sonication and incubation. The AA-PEG amount was optimized in order to achieve maximal DL, which resulted in about 33% (amount of the drug/amount of EXO proteins). Western blot analysis confirmed that the preparation procedure did not affect EXO protein content, while DLS analysis revealed increased average size and zeta potential of AA-PEG-EXO-PTX (304 nm and −4.4 mV), compared to plain EXOs and PTX-EXOs. DiI-labeled AA-PEG-EXO-PTX were uptaken by murine lung cancer cells more than the controls, evidence also confirmed by the ex vivo observation of the co-localization of AA-PEG-EXO-PTX with pulmonary metastases in lung cancer-bearing mice, with consequent increased therapeutic efficacy.

In addition, Wang and his group prepared PTX-loaded EXOs isolated from murine macrophages [74]. In particular, through sequential centrifugations, the EXOs were isolated from lipopolysaccharide-activated M1 macrophages, able to produce EXOs with documented antitumor activity [113]. PTX was loaded through sonication followed by incubation at 37 °C, to restore the EXO membranes, obtaining a DL of 19%. DLS measurements revealed an increased size of 173 nm for PTX-EXOs, compared to plain EXOs and sonicated EXOs, probably due to the partial adsorption of PTX to the exosomal membranes, while TEM observation showed that no morphological changes occurred after sonication and PTX loading. An MTT assay performed on 4T1 breast cancer cells evidenced an anti-proliferative effect of PTX-EXOs, also confirmed by a flow cytometry analysis detecting a higher number of apoptotic cells after treatment with PTX-EXOs, compared to the controls. An in vivo test on mice xenografted with 4T1 cells evidenced reduced tumor volume and prolonged survival in those animals receiving PTX-EXOs, compared to the controls, which were treated with plain EXOs and free PTX.

An alternative source of EXOs is represented by cow milk: indeed, Agrawal and coworkers proposed milk-derived EXOs as oral carriers of PTX [75]. EXOs were isolated from raw milk using a sequential centrifugation procedure [114], and PTX was loaded by incubation. PTX loading caused an increase in EXO size (from 75 to 108 nm), zeta potential (from −15 to −7 mV), and polydispersity index (PDI, from 0.15 to 0.19), determined by DLS; these changes might be due, as stated by the authors, to the partial absorption of PTX to the vesicle surface through hydrophobic interactions. Unfortunately, the DL, measured by ultra-performance liquid chromatography (UPLC), was just 8%. The stability of the formulation was confirmed by monitoring the particle size in simulated gastrointestinal fluids, with a PTX release of about 20% after 2 h, increasing to 40% after 4 h; importantly, the drug release was not influenced by the different pH of the media used in the assay. The PTX-EXOs yielded higher tumor-growth inhibition and lower systemic toxicity in lung cancer-bearing mice, compared to plain EXOs and free PTX.

Similarly, Kumar and his group developed PTX-loaded EXOs deriving from bovine milk [76] for oral delivery, but they also functionalized the vesicles with folic acid (FA), considering the overexpression of folic acid receptor in tumor cells [115]. After isolating the EXOs from raw milk through sequential centrifugations, the active was loaded by sonication (in this case not followed by incubation at 37 °C); FA was linked to the vesicle surface through EDC coupling. The final FA-PTX-EXOs exhibited, through DLS, an average size of 95 nm, negative zeta potential (−27 mV) and low PDI (0.158). Encapsulation efficiency (EE) and DL, determined by UV spectrophotometry, were 82% and 26%, respectively; scanning electron microscopy (SEM) evidenced the spherical morphology of the final vesicles. Interestingly, the authors froze and lyophilized the FA-PTX-EXOs, after the addition of trehalose as a cryoprotectant, and the reconstituted vesicles exhibited no significant changes in size and drug content. The formulation showed a burst release of PTX (25%) in PBS medium in the first hour, followed by a sustained release for up to 48 h. Confocal microscopy revealed that FA-functionalized EXOs encapsulating the fluorescent coumarin-6 internalized in MDA-MB-231 human breast cancer cells (overexpressing the FA receptor) and localized within the lysosomes. The MTT assay allowed the determination of FA-PTX-EXOs IC50, lower than the one of free PTX and PTX-EXOs; moreover, the annexin-V assay proved the apoptotic inductive effect of FA-PTX-EXOs, also accompanied by the reduction in cell migration ability, investigated using a transwell migration assay. Unfortunately, an in vivo assay proving the gastro-intestinal absorption of the nanosystem was not reported by the authors.

To target glioblastoma, Salarpour and her group isolated human glioblastoma-derived EXOs, using a commercial purification kit, and loaded them with PTX, either by incubation or sonication [77]. DLS measurements revealed that the PTX-EXOs had an average size of 63 nm and 86 nm by following the incubation and sonication procedures, respectively, with negative zeta potential (between −18 and −22 mV). TEM and SEM confirmed the spherical morphology of the vesicles and, using a commercial assay, the presence of the CD9 tetraspanin surface protein biomarker was confirmed. Unfortunately, PTX loading, determined by HPLC, resulted in only 0.74% for incubation and 0.92% for the sonication method. The MTT assay confirmed higher glioblastoma cell growth inhibition of PTX-EXOs, compared to free PTX and plain EXOs.

Differently from the abovementioned papers, some researchers have been dedicated to the development of taxane-loaded bio-inspired particles, mimicking the EVs in their structure and homing abilities. In these research works, various bioderived membranes, obtained either from healthy or from cancer cells, have been tested on several types of synthetic NPs.

A widely investigated strategy is to develop red blood cell (RBC)-mimicking nanosystems by covering different kinds of NPs with RBC membranes, providing the nanosystem with biocompatibility and protecting it from circulation clearance; moreover, RBCs are an abundant source of biomembranes and, lacking internal organelles, their workup is easy.

In this context, Su et al. [78] proposed polymeric NPs composed of a core of poly(caprolactone) (PCL) and encapsulating PTX, coated with RBC membranes. RBCs were isolated from heparinized mouse blood and membrane fragments were obtained after sequential centrifugations; then, RBC membranes were resuspended, sonicated and extruded through 400 nm and 200 nm polycarbonate filters. The core NPs were prepared through the nanoprecipitation method by adding dropwise a PCL and PTX acetone solution to an aqueous solution of Poloxamer 188, then the mixture was homogenized and centrifuged. The RBC membrane coating was applied by co-extrusion, obtaining the final RVNPs. DLS measurements evidenced increased average size after the coating (from 134 to 148 nm), while the negative zeta potential remained substantially unchanged; TEM micrographs confirmed the effective covering of the NPs with the membranes. The DL and EE of RVPNs, probably determined by HPLC (the analytical method combined with dialysis for determining the drug release profile), were 4.0% and 95.7%, respectively; the coated particles showed a slower release profile compared to the uncoated ones. The immune escape ability of the RVNPs was assessed in vitro by incubating the nanosystem with mouse macrophages and observing scarce internalization using confocal fluorescence laser scanning microscopy and flow cytometry. In vitro studies on murine breast cancer cells revealed an RVNPs internalization comparable to that of the uncovered NPs, and consequently negligibly impaired by the RBC membrane coating; interestingly, the authors observed that the co-administration of RVNPs with the iRGD peptide in SD rats led to remarkably enhanced PTX permanence in blood circulation.

In a following work, the same authors [79], to overcome the drawbacks related to their RBC-coated nanosystem, including the lack of targeting ability and the difficulty of PTX release from the NPs due to the presence of intact RBC membranes forming a barrier, integrated the near infra-red (NIR) laser-responsive 1,1-dioctadecyl-3,3,3,3-tetramethylindotricarbocyanine iodide (DiR) into their RBC-mimetic NPs. Indeed, irradiation of an NIR photosensitizer with a laser of 650–900 nm might enhance tumor penetration with remote and precise control [116]. The RBC-coated NPs (PTX-PN@DiR-RV) were prepared by co-extrusion of the DiR-labeled RBC membranes with the polymeric core, which included also the thermosensitive phospholipid 1,2-dipalmitoyl-sn-glycero-3-phosphocholine (DPPC): indeed, this lipid undergoes a thermal transition triggered by the DiR absorption of an NIR radiation and thermal energy release, leading to the destruction of the polymeric core and the consequent release of the loaded drug. DLS measurement of the final coated NPs revealed an average hydrodynamic diameter of 151 nm and a zeta potential of −14 mV; the DLs of DiR and PTX were 10.1% and 4.1%, respectively. After observing the enhanced PTX release in PBS upon NIR stimulation of the coated NPs, the authors evidenced that laser radiation enhanced the cellular uptake of fluorescently-labeled PTX in murine breast cancer cells treated with PTX-PN@DiR-RV, accompanied also by a reduced cell viability, determined by a sulforhodamine B staining assay. Ex vivo and in vivo studies on breast cancer-bearing mice revealed radiation-enhanced peritumoral accumulation of PTX-PN@DiR-RV, with PTX concentrations 2.1-, 2.4-, and 2.3-fold higher than those without irradiation at 4.5, 8, and 24 h post-injection, respectively, correlated with significant tumor-growth suppression.

**Figure 4:**
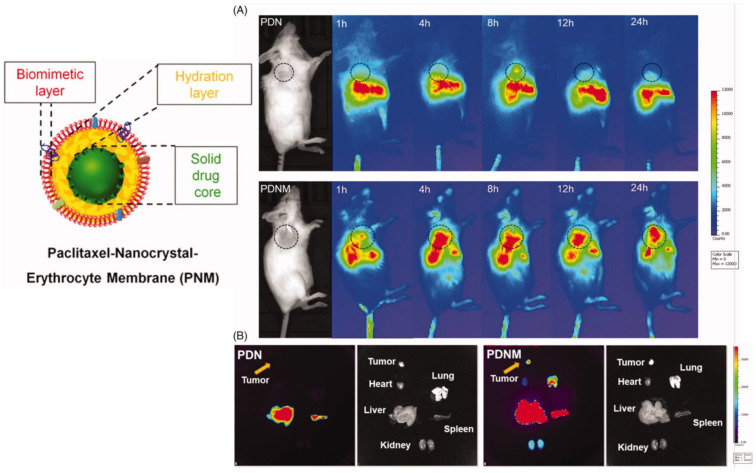
Scheme of EM-coated PEG-PTX NPs and in vivo NIR fluorescence imaging of DiR-labeled uncoated (PDN) and EM-coated (PDNM) PEG-PTX NPs. (**A**) In vivo imaging of 4T1 cancer-bearing BALB/c mice receiving a single injection of PDN or PDNM, respectively. The dashed black circles indicate the tumor burden. (**B**) Ex vivo imaging of tumor and organs excised from 4T1 tumor-bearing mice 24 h post injection of the two fluorescent formulations. Compared to PDN, the PDNM showed higher drug distributions at the tumor site. The yellow arrows point to the tumor tissue. Reproduced from [80] under the Creative Commons Attribution (CC BY) license (http://creativecommons.org/licenses/by/4.0/ accessed on 28 March 2023).

To improve PTX loading, Zhai et al. [80] proposed biomimetic NPs composed of a core of hydrophobic PTX nanocrystals surrounded by an intermediate amphiphilic layer of polyethylene glycol (PEG)-conjugated PTX, covered with an erythrocyte-mimicking shell (EM). For the synthesis of PEG-PTX, whose structure was confirmed by ^1^H-NMR, a carboxylic moiety was introduced on PEG by a reaction with succinic anhydride and then activated by EDC, and the obtained molecule was reacted with PTX. To investigate the effect of different NPs shape, the authors prepared both rod-shape pure PTX NPs through the film dispersion method and subsequent incubation–sonication procedure, and spherical-shaped NPs using the emulsion-lyophilized crystallization method; then, these NPs were covered with the PTX-PEG layer using probe sonication followed by lyophilization, centrifugation and resuspension. To prepare the EM-coated NPs, PEGylated PTX NPs were mixed and sonicated with the vesicles obtained from murine RBCs through sequential centrifugations, sonication and extrusion. The EM-coating of rod-shaped NPs was not successful, as evidenced by TEM, while EM-coated spherical PEG-PTX NPs showed, through DLS, an average diameter of 327 nm, minimally larger than the uncoated NPs, and a slightly negative zeta potential. Sodium dodecyl sulfate-polyacrylamide gel (SDS-PAGE) analysis confirmed that the final coated NPs retained almost all membrane proteins, compared to the original erythrocyte membranes. PTX loading was over 60%, with slow release in sink conditions. The EM-coated NPs, after labeling of the NP core with the fluorescent DiR dye, exhibited higher uptake and antitumor effect in different cancer cell lines, compared to the uncoated NPs. In vivo imaging of breast cancer-bearing mice evidenced accumulation of the EM-coated NPs at the tumor site, and ex vivo observation of the dissected organs showed an inhibition of tumor growth, in addition to metastasis formation prevention (Figure 4).

Song and coworkers [81] exploited murine RBC membranes for the development of pH-sensitive biomimetic NPs for the vehiculation of PTX. Using ultrasounds (US), the authors prepared a polymeric core composed of carboxymethylcellulose grafted with stearic acid through a carbodiimide crosslinking reaction, confirmed by ^1^H-NMR. The RBC membranes were isolated using sequential centrifugations, sonication and extrusion, and, to achieve cancer targeting ability, 1,2-distearoyl-sn-glycero-3-phosphoethanolamine (DSPE)-PEG-FA was inserted in the membranes by incubation. The thus-obtained vesicles were mixed with the core NPs and subjected to US, and PTX was loaded by incubation (EE = 11.5%), obtaining the final PTX-FRCS NPs. Distinct core–shell spherical structures were observed using TEM, with an average size of 227 nm and zeta potential of −14 mV. PTX-FRCS NPs possessed a protein profile similar to the one of the original erythrocyte membrane, as evidenced by the SDS-PAGE analysis, while fluorescence-activated cell sorting (FACS) analysis demonstrated that the RBC coating prevented PTX-FRCS NPs uptake by murine macrophages. The pH sensitivity was assessed by observing increased PTX release under acidic conditions, simulating the tumor microenvironment, and confocal images highlighted the PTX-FRCS NPs internalization in different cancer cell lines. After observing the in vivo accumulation of PTX-FRCS NPs in mouse tumor xenografts, the authors demonstrated that the administration of PTX-FRCS NPs to mice xenografted with liver cancer, lung cancer and melanoma resulted in suppression of tumor growth.

To target malignant melanoma, Cao et al. [82] proposed PTX loaded-albumin NPs coated with murine macrophage membranes, based on the evidence that macrophage coating enhances tumor targeting [117]. Cell membranes were isolated using hypotonic lysis, followed by mechanical membrane fragmentation, differential centrifugation and extrusion, while albumin NPs were prepared using a nanoprecipitation process. The final membrane-coated albumin NPs (PTX-RANPS), obtained by co-extrusion of the two components, exhibited a hydrodynamic diameter of 189 nm, which is between the size of membrane vesicles and uncoated albumin NPs, and negative zeta potential, as measured by DLS. The effective coating was proved by TEM observation, and PTX EE, determined by LC-MS/MS, was 83.4%. Flow cytometry and confocal laser scanning microscopy performed on different cancer cell lines revealed a significantly higher uptake of PTX-RANPS, compared to the uncoated albumin NPs, possibly following a lysosomal pathway after internalization; moreover, PTX-RANPS exerted higher cell-viability reduction, compared to the controls. Ex vivo fluorescence observation proved tumor-specific distribution and prolonged circulation time of PTX-RANPS in melanoma-bearing mice, accompanied also by a reduction in tumor volume, although not significant if compared to the control animals.

Several research groups, investigating novel effective bioinspired nanosystems for PTX delivery, decided to exploit cancer cell membranes to camouflage artificial NPs. Indeed, cancer cells feature immune escape and homing abilities [118], and are ideal cell membrane sources, since they are easy to culture in vitro.

In this context, to improve the therapeutic effect against cervical cancer, Xu et al. [83] developed cancer cell membrane-camouflaged NPs for the simultaneous delivery of PTX and an siRNA targeting the oncogene E7, which is possibly associated with PTX resistance. In particular, the authors prepared a poly(lactic-co-glycolic) (PLGA) core encapsulating PTX and siRNA, using the double-emulsion solvent evaporation technique, and human cervical-cancer-cell membranes were isolated by sequential centrifugations and extrusion; the final Si/PNPs@HeLa were obtained by co-extrusion. DLS measurements revealed that the membrane coating led to a decrease in zeta potential, from −14 to −30 mV, and a size increase of about 15 nm, while TEM micrographs evidenced a core-shell structure with a spherical shape. The EE of PTX and siRNA, determined by HPLC, were 90.2% and 88.4%, respectively, and gel electrophoresis followed by Coomassie blue staining confirmed that the final NPs retained the membrane proteins characterizing HeLa cells. Quantitative reverse transcription (qRT-PCR) analysis showed a 75% Si/PNPs@HeLa knockdown effect on the E7 oncogene after 48 h, significantly higher than that of the uncoated NPs. In vivo biodistribution using NIR-fluorescence whole body imaging performed on HeLa tumor-bearing mice receiving labeled Si/PNPs@HeLa highlighted increased fluorescence signals in the tumors and reduced non-specific accumulation in healthy organs, compared to uncoated NPs, and the authors confirmed the synergistic action of siRNA-E7 and PTX by observing the smallest tumor volume and the largest necrotic area in mice treated with Si/PNPs@HeLa, compared to the controls.

Similarly, to obtain a targeted nanosystem for treating osteosarcoma, Cai et al. [84] coated PLGA NPs, loaded with PTX, with hybrid membranes obtained from human osteosarcoma (143B) and murine macrophage (RAW264) cell membranes, thus combining the tumor homing properties of the cancer cells with the tumor-escape abilities of macrophages. The PTX-loaded PLGA NPs were prepared using the nano-precipitation method, while cell membranes were isolated by gradient centrifugation and sonication of the original cells [119]. The hybrid membranes were prepared by ultrasonic treatment of a 1:1 dispersion of the two types of membranes, and the final PTX-PLGA@[143B-RAW] NPs were obtained by adding the PTX-loaded PLGA NPs to the hybrid membrane suspension, with ultrasonic treatment selected as the best method, compared to co-extrusion, since it produced NPs with more reproducible sizes and higher membrane protein content. The final NPs had a spherical morphology and a core-shell structure, as evidenced by TEM, with a diameter of about 220 nm, determined by DLS. PTX EE and DL were 64.9% and 4.2%, respectively, and PTX release, evaluated in a PBS buffer, was pH-dependent, increasing at the low pH typical of the tumor microenvironment. In vitro internalization tests on osteosarcoma cells evidenced higher uptake and cytotoxicity of the hybrid membrane-coated NPs; moreover, using TNF-α stimulated HUVEC cells to mimic the inflammatory environment, and the authors proved that the macrophage cell membrane coating could improve the chemotactic effect of the NPs on the inflammatory environment. Then, using a live imaging system, a higher accumulation of the PLGA@[143B-RAW] NPs was observed, and PTX-PLGA@[143B-RAW] NPs induced a higher decrease in tumor volume in osteosarcoma-bearing mice, compared to the controls.

### 2.2. Camptothecin

Camptothecin (CPT) ((S)-4-ethyl-4-hydroxy-1,12-dihydro-4H-2-oxa-6,12a-diaza-dibenzo[b,h]fluorene-3,13-dione) is a monoterpene indole alkaloid characterized by a highly hydrophobic pentacyclic structure, first described in a paper dating back to 1966 by Wall et al., isolated from the bark and stem of *Camptotheca acuminata* (Camptotheca, Happy tree), a rare Chinese tree belonging to the Nyssaceae family used in traditional Chinese medicine as a natural remedy against cancer [120]. Its presence has also been confirmed in *Chonemorpha fragrans*, also known as the frangipani vine, a climbing shrub native to tropical/subtropical Asia, in *Nothapodytes foetida* and *Ervatamia heyeano* [121]. Preclinical and clinical studies were actively conducted in the late 1950s and mid-to-late 1960s, but in the 1970s, in spite of showing anticancer activity, these were interrupted, due to inconsistent results and side effects [122]; only in the late 1980s was the CPT mechanism of action clarified [123]. Several extraction techniques were proposed, and besides the classical Soxhlet and US procedures, microwave-assisted and accelerated solvent extraction have been proposed [124], but, due to the low yields, alternative ways such as synthetic preparation [125] or extraction from cell cultures [126] have been attempted.

CPT is a potent topoisomerase 1 (TOP1) inhibitor, a key enzyme involved in transcription [90]: it can rapidly penetrate the cell membrane and bind and stabilize the TOP1 cleavage complexes with relatively low affinity but high selectivity, paving the way for the generation of toxic double strand DNA breaks, thus reducing the frequency of cells in the S phase of the cell cycle [85]. Notably, TOP1 is its unique target, and only the natural stereoisomer 20S is active.

CPT owes its activity to the intact lactone ring, which rapidly hydrolyzes into the more toxic and less potent carboxylate form in the plasma, where it associates with albumin, being responsible, together with its very low solubility in water, for CPT low bioavailability. Attempts to provide improved properties for this compound have included the synthesis and testing of numerous water-soluble analogues such as irinotecan and topotecan, although featuring lower IC50 [90]. Among them, in 2007 5(S)-(2′-hydroxy ethoxy)-20(S)-camptothecin was designated as an orphan drug for the treatment of osteosarcoma; the same occurred in 2019 for 7-ethyl-10-hydroxy-camptothecin for the treatment of soft tissue sarcoma. In spite of causing serious side effects, such as life-threatening neutropenia and cystitis [89], it is currently an object of renewed interest for the treatment of aggressive cancers such as colon, lung, breast, stomach and ovarian tumors [86]. Indeed, the complexes between CPT and DNA must survive long enough to be converted into DNA damage, but, due to the camptothecin complex instability, the drug must be given as a prolonged infusion to maintain persistent cleavage complexes; on the other hand, strategies to prolong its presence in the body are actively pursued [127].

CPT can be used as a radiosensitizing agent, by reducing the number of cells in the S phase of the cell cycle, when they are more resistant to ionizing radiation. This may be a successful strategy in the treatment of aggressive tumors, such as advanced cervical cancer. The idea of Yang’s group [85] was to isolate vesicles from the patient’s own tumor tissues, exploiting their innate targeting properties. EXOs production was stimulated in plated cells from patient tumor tissue using UV irradiation; later, the vesicles were isolated using ultracentrifugation, and CPT was loaded using electroporation. As a control, CPT-loaded EXOs deriving from HeLa cells were also prepared. The vesicles were characterized for morphology, dimensions (approx. 115 and 120 nm for the patient’s own and HeLa cell-derived EXOs, respectively), loading efficiency (23.5 and 21.5% respectively, assessed using confocal laser scanning microscopy, exploiting the CPT natural fluorescence) and zeta potential (approx. −20 mV, independently of their origin). Vesicle dimensions did not change significantly after UV irradiation, a key feature for the successful application as radiosensitizers. Western blot confirmed the presence of exosomal marker proteins CD9 and CD63 on both types of vesicles. Their cytocompatibility was assessed on several cell lines, and both significantly increased radiotherapy-induced cell apoptosis compared to radiotherapy or CPT treatment alone. As expected, cell viability decreased more when the cell line was treated with self-deriving EXOs. Flow cytometry showed that the treatment with own-derived EXOs reduced the percentage of cells in the S phase. The technology was tested in vivo on patient tissue-xenografted mice: after injection, CPT levels in the tumors of mice treated with patient cell-derived EXOs were 6.1- and 1.9-fold higher that the ones due to free CPT and to CPT loaded into HeLa cell-derived EXOs, respectively; patient-derived EXOs also showed a higher tumor-to-healthy-tissue distribution ratio. A second test in vivo was carried out by injecting free CPT and CPT-loaded EXOs, followed by radiation therapy: the host’s own cell-derived vesicles revealed high antitumor activity (94% reduced tumor volume vs. control); moreover, the EXOs prolonged CPT circulation time and were devoid of off-target toxicity. This technology, although difficult to apply in real clinics, offers a notable example of efficient personalized therapy in cancer treatment.

Besides loading CPT into natural EXOs, for PTX it is possible to use biologic membranes either to prepare synthetic vesicles or as coating of synthetic NPs.

Hsieh et al. [86] conceived combining RBC-derived biovesicles with the concept of triggerable devices, whose drug delivery may be induced by external stimuli such as US, being non-invasive and highly penetrating. An RBC ghost pellet obtained from the blood of C57BL mice was suspended in PBS containing glycerol and perfluoro-n-pentane; the mixture, once vortexed and sonicated, generated microdroplets of the organic phase immediately, covered with an RBC membrane shell; drugged droplets were obtained by including CPT in the initial mixture. Using an electrical sensing zone system, the droplets exhibited a size of approx. 1.7 µm and a low PDI. Gel electrophoresis analysis proved the presence of membrane proteins on the final particles, but a significant protein loss from the droplets occurred after 4-day storage at 4 °C, even in the presence of a protease inhibitor; Western blot evidenced that after 10-day storage the droplets lost CD47, which might play a key role in the suppression of macrophage phagocytic activity. CPT loading efficiency and EE, ranging from 0.9 to 3.4% and from 102.6 to 80.9%, respectively, were determined by exploiting CPT natural fluorescence. Flow cytometry showed that, interestingly, freshly prepared RBC-derived droplets showed very low uptake into primary mouse peritoneal macrophages (13.5%) if compared to 10-day-old droplets (56.6%), a result probably related to the aforementioned protein composition change, and slightly lower than that of synthetic PEGylated acoustic droplets (25.1%), demonstrating that the RBC-derived vesicles prevent phagocytosis and may be applied as long-circulating vehicles, without the necessity of PEGylation; nonetheless, the similar slow uptake of the synthetic droplets might make the advantages of using this technology dubious. In addition, the ageing of the droplets notably affects their stealth properties, and this might impair their successful application in therapy. B-mode US imaging was used to evaluate the US contrast-enhancing ability of the droplets before and after acoustic vaporization, confirming their potential suitability for cancer imaging. An MTT assay on HeLa and human lymphoblastoid cells highlighted the fact that the viability remained high when the cells were treated with US alone, unloaded and CPT-loaded droplets, while it dropped to 50% with unloaded droplets+US (suggesting that droplet vaporization might physically damage the cells); finally, the drugged droplets resulted in a release of 40% of the loaded CPT upon insonation, causing a viability reduction of 30%. In vivo tests were performed on mice injected with the drugged RBC-derived vesicles and then treated with US on the right leg venous sinus: a US contrast enhancement, indicative of the presence of bubbles, was immediately detected in the treated leg, confirming droplet vaporization occurrence. However, it is unclear whether these droplets would be uptaken by healthy cells; moreover, the authors themselves acknowledge, differently from other research groups using RBCs, that the necessity of sterile biologic starting material is a limit to this technology; also, blood-type matching is necessary in the case of non-autologous origin.

A similar formulation was also proposed by Ghasemzadeh and colleagues [87], who used RBCs for the preparation of nanobubbles with a sulfur hexafluoride core. They observed that theranostic nanobubbles are rapidly cleared from the blood by kidneys and the liver, thus sparingly accumulating into the tumor masses. Therefore, they conceived the idea of decorating the surface of RBC-derived nanobubbles with an aptamer developed against MUC1, a glycoprotein overexpressed in several types of cancer, including colorectal cancer. In addition, in this case, RBC-derived vesicles were obtained from mice blood using centrifugation and sonication, and CPT loading was performed using incubation. DL and EE were measured spectrophotometrically (79.2% and 7.9%, respectively). For active targeting, the aptamer, whose terminal carboxylic group had been previously activated with EDC/NHS, was incubated with the loaded nanobubbles, and the successful binding was confirmed using agarose gel electrophoresis. Dimensions and zeta potential of undecorated and decorated nanobubbles were determined by DLS (349 and 478 nm, −2 and −8 mV, respectively). CPT release was measured spectrophotometrically over 9 days, using dialysis: apparently, 41% of the loaded CPT was released at the end of the study, but the authors do not propose any justification for the missing 59%; however, they noted that in vivo, in the presence of hydrolases, the release was complete. An MTT assay performed on mouse colon carcinoma cells and on Chinese hamster ovary cells (MUC1 positive and MUC1 negative, respectively), evidenced that the targeted vesicles resulted more cytotoxic on the cancer cells, while no significant difference was found in targeted and untargeted vesicle cytotoxicity on the healthy cells; in all cases, free CPT cytotoxicity was higher than that of the vesicle, as the free molecule can probably enter the cells via diffusion rather than endocytosis. To visualize the vesicles within the cells through fluorescence microscopy, the nanobubbles were loaded with DiI (unusually, the authors did not exploit the CPT natural fluorescence). It has to be noted that this work also evaluated the uptake by normal cells, which was not considered in the other works; unfortunately, the formulation, even the targeted one, resulted in being noxious also to normal cells, and this aspect should be considered in the perspective of long treatments. BALB/c mice subcutaneously injected with C26 cells and receiving the CPT-loaded nanobubbles showed no significant weight loss, significantly lower tumor growth and a higher survival rate compared to mice treated with the free drug, with better performance by the targeted vesicles. Vesicle distribution, evaluated by ex vivo fluorescence imaging on mice treated with DiI-loaded vesicles, revealed significant accumulation in the tumor, and also in this case, a better performance by the targeted ones; US imaging was also assessed 3, 5 and 10 min after administration, showing increasing echogenicity, which was attributed by the authors to progressive tumor accumulation of the vesicles. Unfortunately, the measurement was performed only on a mouse receiving untargeted vesicles, so no beneficial effect of targeting could be highlighted. One of the limits of this research is that the loading capacity of the final nanobubbles has not been measured.

Malhotra and colleagues [88] used CPT as a model of a hydrophobic drug to study the incorporation and retention in RBC membranes, but in this case human blood was used as the starting material. In addition, the procedure for vesicle preparation was different, as after hemolysis the erythrocytes were resealed and then extruded through 0.4 and 0.2 µm polycarbonate filters. For drug loading, resealed RBC were suspended in a hydroalcoholic solution of CPT and the fluorescent dye CM-DiI, and were incubated prior to washings and extrusion. The authors declare that the extrusion process is superior to sonication, due to better size reproducibility. Vesicle size and morphology were evaluated by TEM; DLS was employed to measure particle size and zeta potential (approx. 207 nm and −8 mV, respectively), stable even after 24 h incubation at 37 °C. Drug-loaded vesicles resulted in being slightly larger (250 nm), with no significant difference in zeta potential, coherent with the neutral nature of CPT. SDS-PAGE was used to outline protein composition, while an immunoassay confirmed the presence of the membrane protein CD47. CPT loading and release in PBS were quantified through dialysis by exploiting its fluorescence: EE reached a maximum of 85%, and CPT release increased from 5.5% after 1 h to 50 and 80% after 6 and 24 h, respectively. On the contrary, the fluorophore CM-DiI was strongly retained, possibly due to its amphiphilic nature, thus making this system very attractive for theranostic applications. The hemolytic properties of the vesicles, assessed in vitro, were negligible, while an MTT assay on adenocarcinomic human alveolar basal epithelial cells and lung carcinoma cells confirmed, through fluorescence microscopy and flow cytometry, extensive uptake, and a cytotoxicity comparable to free CPT, via apoptosis and not necrosis. Fluorescent vesicle uptake by human monocytes differentiated into macrophages, evaluated by fluorescence microscopy and flow cytometry, confirmed the good stealth properties, to be attributed to the conserved CD47. Undrugged CM-DiI-loaded vesicle compatibility was assessed in vivo on mice: the vesicles showed longer retention in the blood compared to PEGylated liposomes, reaching the maximum concentration in the liver after 24 h, while their amount further increased in the spleen up to 48 h, coherently with the spleen function.

Malhotra et al. [89] also conceived the idea of applying their RBC-derived vesicles to combination therapy, which is based on the association of well-tolerated photosensitizers with chemotherapeutics to achieve a photothermal-chemotherapy synergistic effect, allowing for the administration of lower doses, and hence reducing side effects. This combination requires multifunctional carriers bearing the associated molecules simultaneously to the target. As photosensitizer, they chose indocyanine green (ICG), which, by absorbing light, can generate ROS and cause localized hyperthermia, but whose clinical application is limited by its very short half-life (2.5 min). Unusually, in spite of the results obtained from the hybrid vesicles, the authors employed neat RBC-derived vesicles, prepared as already described [88], this time incubating resealed erythrocytes with CPT and the photosensitizing agent before extrusion. Apart from the size (80–120 nm, similar in unloaded and loaded vesicles) and morphology, zeta potential was measured, being approx. −13 mV for unloaded and loaded vesicles. Interestingly, ICG loading efficiency (determined by absorbance) improved by increasing the concentration, as already noted with CPT, reaching a maximum of 80%. ICG higher polarity, compared to CPT, accounts for the higher affinity of RBC membrane towards the photosensitizer, confirmed also by the observation that 80% ICG was still retained in the vesicles after 48 h. This might be favorable for intratumor application, as the dye would be kept in the vesicle membrane and be protected from degradation. The photodynamic activity of the ICG-loaded vesicles under NIR irradiation was measured, revealing an additive effect of CPT to the photodynamic activity of ICG. In addition, any oxidative degradation of CPT due to ROS formation induced by ICG was ruled out through an HPLC assay. The photothermal properties of the vesicles were established in suspension and in the cell culture upon vesicle uptake; however, the authors acknowledge that vesicle concentration must be optimized to minimize thermal damage to surrounding healthy tissues. The vesicle uptake on lung carcinoma cells, which reached a maximum after 24 h incubation, was 3–4-fold lower, compared to free ICG: this might be explained by the various pathways the free photosensitizer may use to enter the cells. MTT and the apoptosis assay showed that the combination of CPT + ICG + NIR led to approx. 18% residual viability, significantly lower than that resulting from the treatment with vesicles, including CPT or ICG only, confirming the superior efficacy of combination therapy. As in the previous study, Ehrlich Ascites Carcinoma (EAC)-bearing mice were treated with vesicles injected intravenously and intratumorally: as observed previously, 24 h after systemic administration most vesicles were in the liver, but a significant number were in the tumor; when injected into the tumor, the vesicles remained in place for up to 72 h, due to typical tumor-enhanced retention. By administering CPT + ICG vesicles twice intratumorally, followed by NIR irradiation, a sizable 50% growth reduction was reached. ROS formation was also evaluated, confirming that ICG preserves its ROS-generating properties even in vivo.

The same research group [90] tried to enhance CPT solubility, slow its degradation, and improve its overall pharmacokinetic profile by preparing RBC membrane-coated polymeric micelles, which may effectively encapsulate the drug in the polymeric core, minimizing its hydrolysis, while the biologic membrane coating might offer a further barrier for release control. For this purpose, a di-block PEG-PCL polymer was synthesized by ring-opening polymerization and fully characterized, identifying the structure, average molecular weight and a polydispersity index of 1.06; the polymer was then employed for micelle production, via the solvent evaporation method, and CPT was included by simply dissolving it in the organic phase. Then, RBC ghosts, prepared as reported in the previously mentioned paper, were mixed with Nile Red-labelled micelles and extruded through 0.8 and 0.4 µm polycarbonate filters (differently from the aforementioned work). The resulting vesicle size (approx. 150 nm) was slightly higher than that of the neat micelles, and their zeta potential was approx. −7 mV, slightly higher than that of the RBC ghosts, which may suggest a possible loss of membrane constituents, an aspect that would require an in-depth analysis. As expected, by increasing the polymer:drug ratio, CPT EE improved (up to 85%); as for DL, it increased from the 1.9% of the RBC-derived vesicles to 11.6% of the hybrid vesicle-micelles, which thus resulted in a better platform to vehiculate the appropriate amounts of drugs to the tumors: the difference may be due to CPT partitioning in the polymeric core and in the vesicle bilayer coating. The hybrids resulted in being superior to RBC-deriving vesicles also in terms of release control: only 61% of the loaded drug was released in 48 h, likely due to the presence of two diffusional barriers; moreover, the hydrophobic polymeric core interacts more strongly with CPT. A HPLC study revealed that in serum CPT encapsulated into nanovesicles alone undergoes 45% hydrolysis within 1 h, increasing to 80% within 6 h: this information, not present in [88], actually reveals that particles deriving from ghosts cannot protect CPT from hydrolysis and are inadequate for clinical application, due to their slow accumulation into the tumor mass; coated micelles were more efficient in drug preservation, as only 25% underwent hydrolysis within 1 h, and 39% after 30 h. The internalization of fluorescently labeled RBC membrane-coated micelles on human adenocarcinomic epithelial cells was observed by fluorescence microscopy, showing cytotoxic effects comparable to the ones induced by equal amounts of free CPT. The particles were also tested in vivo in an EAC mice model: particle accumulation in the tumor was visible 24 h after administration, and increased until 72 h. On the basis of these results, CPT-loaded nanoparticles or free CPT were administered to tumor-bearing mice: the treatment with hybrid particles caused significantly lower tumor volume increase and weight loss, not only vs. controls but also compared to the more toxic free drug; also, no necrosis was found in the tumors of mice treated with the hybrid vesicles, and their overall survival rate was higher. Finally, RBC-coated micelles were more efficient in preventing metastasis and cancer-induced cardiac cachexia and were less hepatotoxic, although being as toxic as free CPT to the small intestine.

Similarly to Malhotra’s group, Ying and colleagues [91] conceived the idea of coating synthetic particles with membranes of mouse-deriving M1-macrophages, with the aim of exploiting their tumor-tropism and their capacity to penetrate tumor cores lacking blood vessels; a CPT derivative, 7-ethyl-10-hydroxy-camptothecin (SN38), reversibly bound via an ester bond to a biodegradable oligo-ε-caprolactone fragment, was loaded, aiming to obtain the complete release of SN-38, due to the abundance of esterases inside the cells. The prodrug was dissolved in an acetone/ethanol mixture with the other lipids and added dropwise to deionized water, to obtain solid lipid particles (SLP); RAW 264.7 cells stimulated with LPS generated macrophages, whose membranes were isolated through sequential centrifugations and fractionation on a sucrose gradient. An appropriate amount of the membranes was co-extruded with polymeric SN38-loaded SLP through a 0.4 µm polycarbonate filter, to obtain coated particles (mSLP). The size (approx. 90 nm for coated and uncoated particles), PDI (0.1 and 0.15, respectively) and zeta potential (−23 mV for coated particles) were measured using DLS. The protein composition of the coating, assessed using SDS-PAGE and Western blot, was very similar to the parent membranes. Drug release, determined spectrophotometrically during dialysis, was very slow (reaching just 20% in 1 week), while the presence of porcine liver esterase induced a faster release which, however, reached just 71.5% in 1 week. In vitro cytotoxicity was evaluated by a CCK-8 assay after incubation on mouse melanoma and mouse breast cancer cells, in comparison with the approved SN38 prodrug CPT-11 (irinotecan), having improved solubility but lower bioavailability, as a carboxylesterase is required for SN38 release, with the enzymatic conversion rate generally <10% [128]. The resulting IC50 values of SLP and mSLP were approximately two orders of magnitude lower than that of irinotecan but, interestingly, very close to that of free SN38, demonstrating that the esterases effectively release SN38 from the conjugate; when the conjugate was included in the coated lipid particles, its potency was higher on both cell lines. Confocal fluorescence imaging on both cell lines evidenced the interaction of DiI-labelled coated particles with the cell surface, and fluorescence imaging showed the localization of particles within the endo/lysosomes at early time points and diffused intracellular fluorescence after 20 h, proving the endosome-escaping ability, and allowing for clarification that the internalization was mainly due to clathrin-mediated endocytosis (an assay not performed by other researchers). In a pharmacokinetic study performed on Sprague-Dawley rats after IV injection, the nanoformulations with the SN38-polycaprolactone conjugate showed longer circulation time in the blood and higher AUCs in comparison to irinotecan.

Tumor targeting capacity was evaluated in breast cancer-bearing mice, injecting once the nanoparticles loaded with DiR for fluorescence imaging in vivo and ex vivo: more intense signals were detected in the tumors of mice treated with coated particles, and CPT quantitation in the tumor confirmed this qualitative observation; unfortunately, both formulations showed off-target accumulation in the liver and spleen. In addition, the capacity to target spontaneous liver, spleen and lung metastasis was confirmed for coated NPs. In vivo, the nanoparticle treatment substantially arrested tumor growth, differently from the treatment with free irinotecan, with the mice treated with coated particles showing a significantly higher survival rate, and the capacity to prevent metastasis was also observed. A second model of breast cancer, involving the inoculation of mesenchymal-like murine mammary tumor cells, was employed to confirm the abovementioned results and the low toxicity of the coated nanoparticle treatment.

### 2.3. Curcumin

Curcumin (CUR) ((1E,6E)-1,7-bis(4-hydroxy-3-methoxyphenyl)hepta-1,6-diene-3,5-dione), a polyphenol naturally isolated from the rhizome of *Curcuma longa* (Zingiberaceae) [129], is a constituent of the traditional medicine known as turmeric [130], and it has been widely employed in traditional medicine for its antioxidant (at low concentrations), anti-inflammatory, anti-microbial, and metal-chelating activity, and for its protein-binding capacity [131,132,133,134,135,136,137,138]. Indeed, due to its various effects, CUR has been employed in the treatment of different pathologies such as asthma, rheumatoid arthritis, inflammatory bowel disease, neurodegenerative diseases, and metabolic syndrome, and, used alone or in combination with other drugs, might affect different signaling pathways involved in the development of several types of cancer [139,140,141], but its therapeutic use is hampered by its unstable and nonbioavailable nature [142,143]. Among the different strategies used to overcome these issues, particularly innovative is the use of CUR-loaded EXOs and biomimetic vesicles.

In this context, Aqil et al. [92] sensibly increased the bioavailability and therapeutic efficacy of CUR by loading it into bovine milk-derived EXOs, using a monodisperse population of vesicles (PDI 0.19) with a diameter of approx. 84 nm measured by DLS and confirmed with AFM microscopy. A remarkable DL value of 18–24% (quantified by UPLC) was reached by adding an acetonitrile/ethanol solution of the drug to the EXO suspension. The resulting EXO-CUR were stable at −80 °C in terms of size, PDI and drug content; moreover, EXO-CUR showed not only increased tissue bioavailability in rats, compared to free CUR, but also higher anti-inflammatory and antiproliferative activity on several human cancer cell lines. In vivo studies on human cervical cancer-xenografted rats evidenced that EXO-CUR exerted significantly higher inhibition of tumor growth (61%) compared to that of EXOs alone (21%), while free CUR showed no effect.

In addition, González-Sarrías and colleagues [93] aimed to evaluate whether milk-derived EXOs that incorporate CUR or the polyphenol resveratrol (EXO-CUR and EXO-RSV, respectively) can reach the breast cancer tissue, bypassing their metabolism. EXO isolation and vesicle purification after loading (carried out by sonication, electroporation, or passive incubation) in this case also included size exclusion chromatography, and the vesicle protein profile was assessed using Western blot. Particle size (approx. 185 nm), determined by NTA, did not change after polyphenol loading, which reached the highest value when performed by passive incubation. CUR- and RSV-loaded EXOs exerted significant antiproliferative activity on human breast cancer cells, not affected by the presence of ATP-binding cassette inhibitors, which can hamper the anticancer activity of many drugs. EXO-CUR and EXO-RSV, injected in female Sprague–Dawley rats, produced a peak of free CUR and free RSV in the mammary gland a few minutes after administration; neither CUR nor RSV were detected at any time in the mammary tissue when administered as free drugs. The authors declared that they could not match their results with those of Aqil et al. [92], who encapsulated a much higher CUR concentration, as in that case the delivery of CUR was not studied in mammary tissues. They also observed a high decrease in cell survival when assaying non-loaded EXOs, an effect not observed in the present study. Moreover, González-Sarrías’s EXOs were unequivocally identified after detecting specific EXO-associated markers, but other subtypes of extracellular vesicles could be present in the fractions. In addition, the encapsulation process could be improved to facilitate the delivery of higher polyphenol concentrations to the mammary tissue. Specific future actions might include validating the obtained results in breast-cancer-induced animal models.

Jia and colleagues [94] prepared the RGE-modified, super paramagnetic iron oxide nanoparticle (SPION)-, and CUR-loaded EXOs (RGE-EXO-SPION/CUR), with the aim of combining the magnetic fluid hyperthermia (MFH) effect of SPIONs with the antitumor effect of CUR, while increasing the targeting ability of the vesicles by decorating them with a small peptide ligand of NPR-1, a glycoprotein overexpressed by glioma cells. EXOs were isolated from the supernatant of the mouse macrophage cell culture with ultrafiltration and differential centrifugations, and then SPIONs and CUR were loaded through electroporation. EXO decoration was performed by the conjugation of exosomal phosphatidylethanolamine with 4-pentynoic acid by EDC coupling, followed by reaction with RGE-peptide containing an azido group and FITC, whose fluorescence allowed the confirming of conjugation. The presence of membrane proteins on EXO-SPION/CUR was assessed using Western blot and, after decoration and loading, the vesicle size (123 nm) and zeta potential (−24 mV) slightly increased, compared to plain EXOs. TEM confirmed the presence of SPIONs in the vesicles, and their loading was determined by UV-VIS spectrometry. In addition, SPION loading was confirmed by performing Prussian blue staining on human malignant glioblastoma multiforme cells incubated with EXOs+SPIONs physical mixture and loaded EXOs: interestingly, very few SPIONs were detected in the cells after incubation with the physical mixture, while many were found after incubation at 37 °C with the loaded EXOs; on the contrary, no SPIONs were found when the incubations were carried out at 4 °C, indicating that EXO internalization probably occurs via specific transporters. Fluorescence microscopy and flow cytometry evidenced the higher uptake of DiI-labeled EXOs in NRP-1 positive human hepatoma cells, compared to NRP-1 negative cells; moreover, the combined effect of CUR and MFH induced significantly higher cytotoxicity than CUR-EXOs or plain SPIONs on both cell lines, and was more pronounced on the NRP-1 positive cells. In vivo biodistribution studies on glioma-bearing mice evidenced tumor accumulation of CM-DiI-labeled EXO-SPION/CUR, confirming their blood–brain barrier crossing ability; this result was also confirmed by magnetic resonance imaging. Analogously to what emerged from in vitro studies, targeted EXO-SPIONs and targeted EXO-CUR caused a reduction in tumor volume and an increase in percent survival rate, compared to controls, but these positive results were far more remarkable when targeted vesicles, including both CUR and SPIONs, were administered. In parallel, off-target toxicity was evaluated, confirming the benignity of SPIONs and highlighting the fact that CUR side effects were reduced by targeted encapsulation.

Sonodynamic therapy (SDT) is a tumor treatment based on the interaction of US and sonoactive substances (sonosensitizers), which selectively accumulate in tumor cells and, when exposed to focused US, produce the ROS responsible for tumor-cell killing. This approach has been extensively used in cancer therapy, due to its non-invasiveness, low toxicity, and ability to reach deep tissues, but it shows defects related to the low water-solubility of sonosensitizers and to the necessity of alleviating hypoxia in the tumor microenvironment. Nanoparticle modifications of the sonosensitizers have been proposed to reduce hypoxia and to improve mitochondrial targeting and selective cancer-cell accumulation.

Li et al. [95] designed a bionic nano-system (ECaC) by encapsulating mesoporous calcium carbonate nanoparticles (CaC) and CUR as a sonosensitizer into tumor cell-derived EXOs, obtained from murine colon cancer cells. The EXO membrane, besides providing immune evasion ability to CaCO_3_ and improving targeting, increases the release of Ca^2+^ and CUR in the weak acidic environment of the tumor: Ca^2+^ can destroy the mitochondria of the tumor cells, while CUR can act as a Ca^2+^ regulator, inhibiting calcium efflux and stimulating Ca^2+^ release from the endoplasmic reticulum to the cytoplasm. CaC with a uniform size were prepared using the gas diffusion method, starting from CaCl_2_·2H_2_O and ammonium bicarbonate; CUR was incorporated into CaC by simply including it in the mixture. The colon cancer cells were incubated with CaC, and the loaded EXOs (ECaC) were isolated by ultracentrifugation. Erythrocyte membrane-coated CaC including CUR was also prepared by a similar method (RCaC) and CUR loading was confirmed by UV–Vis spectroscopy. TEM was used to evaluate particle morphology and dimensions (approx. 92 nm), while SDS-PAGE confirmed that EXOs and ECaC shared the same proteome. Ca^2+^ release from ECaC, analyzed by dialysis followed by ICP-AES, resulted in being 80% in less than 12 h at pH 5.5. ECaC exhibited good targeting ability on the parent cells, differently from RCaC, and when combined with US caused conspicuous ROS production that can be attributed to CUR promoting oxidative stress and inhibiting the efflux of Ca^2+^, whose excess can further favor mitochondria destruction. CUR acted also as a sonosensitizer, to achieve an excellent SDT effect, confirmed by the results of an MTT assay. The EXO membrane also enables ECaC to evade the uptake by murine macrophages and this, according to the authors, may prove that this technology might avoid the massive enrichment of ECaC in normal organs, reducing long-term systemic toxicity and toxic side effects. In vivo experiments were performed on colon carcinoma-bearing mice, revealing that ECaC administration, followed by US, caused the maximal tumor suppressor effect and the highest level of tumor cell apoptosis vs. controls, while few ECaC were found in the liver and kidneys, indicating that this system could not cause acute damage to the body.

## 3. EXOs and Biomimetic Vesicles Loaded with Other Phytochemicals

### 3.1. Vincristine

Vincristine (VIN) ((3aR,3a1R,4R,5S,5aR,10bR)-methyl 4-acetoxy-3a-ethyl-9-((5S,7S,9S)-5-ethyl-5-hydroxy-9-(methoxycarbonyl)-2,4,5,6,7,8,9,10-octahydro-1H-3,7-methano [1]azacycloundecino [5,4-b]indol-9-yl)-6-formyl-5-hydroxy-8-methoxy-3a,3a1,4,5,5a,6,11,12-octahydro-1H-indolizino [8,1-cd]carbazole-5-carboxylate) is a vinca alkaloid obtained from the Madagascar periwinkle *Catharanthus roseus*, employed as a medicinal herb in Ayurveda and in traditional Chinese medicine [144]. Vinca alkaloids were first isolated in 1950, but only 10 years later vincristine and vinblastine were approved as anticancer agents by the FDA. Since then, several semisynthetic molecules such as vindesine have been produced and applied in cancer treatment. VIN is active in hematological and solid tumors, such as breast and colorectal cancer, binding to the tubulin proteins in the mitotic spindle, stopping their polymerization into microtubules, and causing the cell to be unable to separate its chromosomes during the metaphase; the cell then undergoes apoptosis, which unfortunately affects all proliferating cells. Another drawback is the P-gp-mediated drug resistance, which causes very limited access of VIN into the tumor cells; for this reason, it is mainly employed in combination therapies. Finally, VIN application is limited by the severe peripheral neuropathy it causes. To improve its therapeutic potential and reduce its side effects, many delivery strategies have been proposed.

A very interesting research study carried out by Del Fattore et al. [96] started from the observation that the scientific literature on MSCs shows a discrepancy in the role they play in tumor growth, either promoting or inhibiting it, and that MSC EVs secretion might take part in their paracrine signaling. Therefore, they isolated neat EVs, using an optimized ultracentrifugation procedure, from three different MSC types (human bone marrow-BM, umbilical cord-UC and adipose tissue-AT), and characterized them for size distribution and concentration using tunable resistive pulse sensing. EVs loaded with VIN were obtained by the incubation of UC MSC cultures with the drug before EV collection. Neat EVs, labeled with the PKH26 fluorescent dye, resulted to associate to U87MG human glioblastoma cells, as seen through confocal microscopy and FACS, and the amount of internalized VIN was determined through a sensitive LC-MS/MS method. The significance of this study resided in the fact that BM or UC-derived EVs reduced the proliferation rate by inducing apoptosis, as evidenced by the FACS analysis, while AT-MSC EV stimulated cell proliferation. The authors acknowledge the limits of these findings, as the tumor cells were cultured in an FBS-free medium and the dose–response relationship was not investigated; also, the physiological relevance of the experimental conditions applied is difficult to assess. Nonetheless, the fact that EVs might be uptaken by healthy cells in vivo is a drawback, suggesting that local injection might be the preferred administration method.

### 3.2. Chrysin

Among the numerous plant-derived compounds with anti-cancer activity is chrysin (5,7-dihydroxyflavone), a natural flavonoid found in honey, propolis, and a variety of plant extracts (*Passiflora caerulea*, *Passiflora incarnata*, *Oroxylum inolicum*) [145,146]. Chrysin is a potent antioxidant and anti-inflammatory agent, and a strong aromatase inhibitor, and has shown cancer chemopreventive activity via the induction of apoptosis in a diverse range of human and rat cell types [147]. Chrysin is poorly soluble in water, with a consequent low bioavailability [148], and hence innovative formulations have been investigated to vehiculate it. For example, Yang et al. [97] proposed chrysin-loaded EVs, isolated by sequential centrifugations from SCC9 human tongue squamous carcinoma cells previously incubated with chrysin. Then, surprisingly, the chrysin-EVs were coated with Au NPs by overnight incubation with HAuCl_4_, to combine the chemotherapeutic potential of the formulation with photodynamic therapy applications. TEM micrographs confirmed the round shape of the final vesicles, which exhibited a size ranging from 50 to 150 nm, measured by DLS. Using confocal microscopy, the authors demonstrated the uptake of the Au-chrysin-EVs on SCC9 cells, even if the Au NPs coating might have affected the homing abilities of the EVs, and NIR irradiation enhanced cell apoptosis, as observed by the TUNEL assay, an effect possibly caused by let-7a-3p gene upregulation, revealed by quantitative real-time PCR. Finally, through in vivo fluorescence imaging, the authors demonstrated that Au-chrysin EVs combined with NIR significantly inhibited tumor growth in tongue squamous carcinoma-bearing mice.

### 3.3. Delphinidin

Delphinidin (2-(3,4,5-trihydroxyphenyl)chromenylium-3,5,7-triol) is an anthocyanin flavonoid present in beans, berries, fruits, and flowers [149], featuring promising cell proliferation inhibiting activity [150,151,152], but it degrades rapidly under physiological conditions since it is light-sensitive and stable only at pH < 3. To overcome such problems, Barkallah and his group [98] encapsulated delphinidin by incubation in EVs isolated from murine dendritic cells through sequential centrifugations. The delphinidin-loaded EVs, with an average diameter of 112 nm (determined by NTA), did not exhibit a size increase compared to the empty vesicles, and the EE, obtained by measuring delphinidin absorbance at 530 nm, was 9%. In vitro tests on human aortic endothelial cells evidenced that, when formulated in EVs, delphinidin was 2-, 10- and 100-fold more potent than free delphinidin in the inhibition of endothelial proliferation, endothelial nitric oxide production and capillary-like formation.

### 3.4. Berberine

Berberine (BRB) (9,10-dimethoxy-7,8,13,13a-tetradehydro-2H-[1,3]dioxolo [4′,5′:2,3]berbin-7-ium) is a natural nonbasic, quaternary benzylisoquinoline alkaloid present in the root, rhizome and stem bark of Chinese medicinal plants such as *Berberis vulgaris* (barberry), *Berberis aquifolium* (Oregon grape), *Berberis aristate* (tree turmeric), and *Tinospora cordifolia* [99,153]. It displays free-radical scavenging property, and its immunomodulatory and anti-inflammatory action makes it a potent neuroprotective agent, besides increasing cell sensitivity to doxorubicin in lung cancer and enhancing apoptosis in HeLa cells, when administered in combination with cisplatin [154]. Unfortunately, BRB is affected by low solubility, scarce GI absorption, and rapid degradation, causing poor bioavailability (apparently <1%); moreover, BRB is a substrate of P-gp, which further limits its clinical outcomes [155]. However, it may easily pass the blood–brain barrier and be slowly eliminated from the central nervous system, which may represent an interesting feature in the case of applications in cerebral tumors.

Salek et al. [99] employed a murine dendritic cell line and isolated their small EVs (sEVs) by serial centrifugations; BRB was loaded by impregnation. Oddly, the vesicles were prepared from a murine cell line and tested on human cells, but, in the discussion section, the authors state that they used sEVs derived from immature human dendritic cells to exclude immunogenicity. BRB loading (3.57 µg BRB/µg sEV, probably a typo) and EE (42%) were determined spectrophotometrically. The size of the loaded vesicles (determined by DLS and NTA) was quite close to that of the native EVs (110 nm and 101 nm, respectively), while the zeta potentials were −5.4 and −11.3 mV, respectively, adequate for a stable suspension. Western blot analysis allowed the identification of the proteins on both unloaded and loaded EVs, showing no significant differences, and the absence of beta-actin, characteristic of large EVs. For the proliferation assay, human breast cancer cells and HUVECs were chosen to represent the complexity of a tumor mass undergoing neoangiogenesis, and the results revealed that IC50 of loaded sEVs was significantly lower than that of free BRB on both cell lines. Interestingly, empty sEVs at high concentrations slightly reduced cell growth: this result was not deeply investigated, but deserved further studies. Both cell types were used for a wound healing assay (mimicking cancer cell migration, as declared by the authors), in which loaded vesicles reduced cell migration more than the free drug in both cell types. The capacity of HUVECs to form capillaries was significantly reduced after loaded sEV treatment; also, they reduced the release of NO, which plays a central role in angiogenesis. This work lacks in vivo tests to confirm the appropriateness of these vehicles for BRB improved efficacy.

### 3.5. Black Bean Extracts

Black bean extracts have revealed antiproliferative activity in vitro in prostate, liver, mammary, and colon cancer cells, possibly due to their content in saponins and flavonoids, which might be involved in cell growth inhibition [156,157]. Unfortunately, the pharmacokinetic profile of plant secondary metabolites is frequently unfavorable.

Donoso-Quezada and coworkers [100] conceived the idea of using EXOs derived from four different cancer cell lines to verify their ability to improve the bioactivity of extracts of black bean seed coats. The extracts were first characterized for their composition by HPLC-DAD-ELSD, revealing the predominant presence of group B soyasaponins and myricetin. EXOs were isolated from human hormone-dependent mammary (MCF7), prostate (PC3), colon (Caco2), and liver (HepG2) cancer cells using a commercial kit, and the total protein content in each sample was determined spectrophotometrically. The size (ranging from 92 to 109 nm, with no significant differences among EXOs deriving from different cell lines) and zeta potential (between −19 and −11 mV) were determined by DLS, and vesicular-shaped morphology by SEM; the typical exosomal marker CD63, detected with a commercial kit, allowed the estimation of the number of EXOs in the samples. Extract loading, performed either by incubation or electroporation, reached the highest value when the second method was applied. The uptake of Bodipy TR Ceramide-labeled EXOs, measured through a microplate reader, started just after 30–40 min, and reached a maximum after 12 h incubation. Using a standard proliferation assay, the extract alone showed powerful antiproliferative activity, in particular on MCF7 cells, but exosomal delivery resulted in a significantly higher effect. It has to be noted that empty EXOs caused higher proliferation compared to the negative control: however, the residual genetic material, whose presence in the cancer-cell-derived EXOs was not measured, might inhibit apoptosis.

### 3.6. Anthocyanidins

Several research articles state the anticancer activity of dietary berries, attributed to their content of anthocyanins [158,159,160]. Unfortunately, these molecules have poor bioavailability when administered orally, as they are scarcely diffusive. Therefore, to be absorbed into the gastrointestinal tract, they must exploit transporters or be hydrolyzed to their aglycones, anthocyanidins (ANTs).

Munagala et al. [101] suggested incorporating ANTs into EXOs obtained from milk, which might be administered per os. The ANTs were isolated from anthocyanin-enriched bilberry extract through a patented extraction procedure. The EXOs were obtained from bovine milk by differential centrifugation, and their total protein content was measured by bicinchoninic acid assay; the loading was carried out by incubation, with a DL of approx. 20%, measured by UPLC. Raw and loaded EXOs were analyzed for size by DLS and AFM (79 and 83 nm, respectively, by DLS; slightly smaller by AFM). The antiproliferative activity was tested in vitro on human lung cancer, breast cancer, pancreatic, prostate, colon and ovarian cell lines using the MTT assay. Interestingly, the EXOs alone caused 8–47% cell growth inhibition, but no hypothesis justifying this activity was proposed; loaded EXOs manifested a 4–60-fold decrease in IC50 values compared to free ANT mixture, a result the authors attributed to better stability and higher uptake, which unfortunately was not confirmed by further, more specific studies. After treatment, nuclear extracts from two cell lines were tested, evidencing higher dose-dependent inhibition of NF-kB activity for ANT-loaded vesicles compared to the free ANT mixture. No sign of toxicity of the ANT mixture was observed on mice receiving ANT by oral gavage. The loaded EXOs induced significantly lower tumor growth in lung tumor-xenografted mice, compared to controls, while the ANT mixture was ineffective.

In another publication [102], the authors focused on the application of extract-loaded EXOs in ovarian cancer. In this case the presence of the typical CD63 marker on EXOs was confirmed by Western blot before loading. The ANT mixture and the loaded EXOs were tested on four different human ovarian cancer cell lines: the ANT mixture alone proved to be antiproliferative, but its effect significantly improved when incorporated into EXOs (which lowered the IC50 by approx. 20 times), a result the authors ascribe to ANT stabilization and better cell uptake. Again, also empty milk-derived EXOs showed a mild antiproliferative activity. Moreover, the ANT mixture increased sensitivity to cisplatin on one cell line when combined with PTX, possibly by decreasing P-gp expression, responsible for drug resistance. In vivo tests were performed on ovarian cancer-xenografted female mice, treated by oral gavage: the ANT-loaded EXOs were more potent in inhibiting tumor growth than the ANT mixture, but the mechanism was not clarified. ANT-loaded vesicles in combination with PTX-loaded ones showed a 78% tumor growth inhibition, much higher than the inhibition obtained by administering PTX-loaded EXOs alone, confirming a conspicuous synergistic effect of the ANT formulation.

## 4. Conclusions

Many natural compounds found in diets, termed phytochemicals, are now used to treat or prevent cancer, but their major drawback is often represented by poor bioavailability, which can be overcome by encapsulation into nanovehicles. Among nanovehicles, biological membrane-derived ones may be preferred for several advantages, against synthetic ones.

One of the advantages of biological carriers is represented by their low immunogenicity, if derived from autologous materials, from which they inherit surface proteins and antigens; this may overcome the limits of synthetic NPs, whose circulating half-life is often increased by PEG coatings that may trigger an immune response causing side effects, even severe, in some patients, while reducing the effect of subsequent administrations by impeding tumor uptake [87,88,91]. Moreover, polymer-based delivery systems offer the advantage of easy ligand linking, but their expensive production and the possible toxicity after long-term use may represent a limit to clinical application on a large scale [102].

The use of EVs, however, might be evaluated with particular care, especially considering the application of tumor cell-derived EVs as drug vehicles. In fact, some experimental evidence suggests that EXOs might enhance tumor cell resistance to anticancer agents, as they might up-regulate the expression of factors, for example, ion channels such as CLIC1, which are related to the reduced activity of anticancer agents such as VIN. This depends on the fact that exosomal expression of these factors is consistent with that of the parent cells, and they might transfer these factors to the receiving cells [161]. Experimental data confirm that EXOs, at least in vitro, if not deprived of their genetic material content, may favor cancer cell survival: this stresses the importance of accurate purification and the absence of nucleic acid verification before employing cancer-cell deriving vesicles for drug delivery. However, this result has to be confirmed in vivo, as many mechanisms are associated with EV interaction with either healthy or cancer cells.

This concern may be overcome by using materials deriving from other biological sources, such as bovine milk or RBC, once the absence of any immunologic issue and toxicity on healthy cells is confirmed. Nevertheless, RBC-derived vesicles should be the object of deeper investigation regarding their interaction with the immune system, as it has been reported that erythrocyte-derived particles injected into healthy female mice induce higher levels of IL-6, IL-10, TNF-α and MCP-1 compared to PBS [162], even though high levels of cytokines do not necessarily mean immune toxicity [90].

Another advantage of biological vehicles may be the intrinsic stealth and targeting properties. From this perspective, biological carriers may be exploited not only to directly encapsulate drugs, but also as a coating of other synthetic NPs, and evidence confirms that, at least in the case of RBC membranes, they cover the particles in a right-side-out manner, preserving the immune escape ability [163].

However, any manipulation applied to biological membranes must be followed by a Western blot or other assay to evaluate any change in protein composition, which might cause the loss of targeting or stealth properties. In particular, the presence of CD47 in RBC-derived vesicles must be preserved, as it confers the ability to escape phagocytosis, due to its interaction with the signal regulatory protein alpha (SIRP α) receptor expressed on macrophages of the reticuloendothelial system [164].

The results gained so far by encapsulating drug molecules into EVs or hybrid particles are valuable, especially in the case of CPT, not only for the targeting ability of the nanovehicles, but also for their stabilizing action against hydrolysis. These preliminary data encourage the researchers to attempt the loading of various actives, not necessarily from a natural origin, into natural or bioinspired vesicles, as these transporters might successfully allow the overcoming of the issues of low stability affecting many active substances currently being developed. However, for some organs, and specifically the small intestine, the toxicity appears comparable to that of the free drug, and this surprising result deserves in-depth studies.

Finally, it must be pointed out that the protocols for evaluating cell uptake and cell cytotoxicity applied by the cited research groups are different, in particular in terms of incubation duration. This makes the comparison between uptake efficiencies difficult.

Moreover, it is important to consider that, since guidelines are still missing for EV formulations, a lack in uniformity in EV size classification and characterization is still evident, and the challenges in the development of efficient GMP protocols for EV isolation and characterization are still to be solved.

## Figures and Tables

**Table 1:** Cell-derived systems classified on the basis of the vehiculated plant-derived active.

Ref.	Source	Plant-Derived Active	Carrier	Isolation Technique/Preparation Procedure	Loading Technique	Functionalization/Engineering	Assays
[70]	*Taxus brevifolia* (Taxaceae)	Paclitaxel	EXOs from SR4987 murine mesenchymal stem cells	Differential centrifugations	Priming of the parental cells with the drug	/	In vitro: CFPAC-1 human pancreatic adenocarcinoma cells
[71]			EXOs from primary human mesenchymal stem cells from umbilical cord	Sequential centrifugations	Priming of the parental cells with the drug	/	In vitro: A549 lung cancer cells, SK-OV-3 ovarian cancer cells, MDA-hyb1 breast cancer cells; ex vivo: female NOD scid mice injected with MDA-hyb1 cells
[72,73]			EXOs from RAW 264.7 murine macrophages	ExoQuick-TC™ Kit	Incubation/electroporation/ sonication	Aminoethylanisamide-polyethylene glycol (AA-PEG) targeting the sigma receptor	In vitro: murine Lewis lung carcinoma cell subline (3LL-M27), Madin-Darby canine kidney (MDCK) cells; ex vivo, in vivo: C57BL/6 mice injected with 3LL-M27 cells
[74]			EXOs from RAW 264.7 murine macrophages	Sequential centrifugations	Sonication	/	In vitro: MDA-MB-231, MCF-7, 4T1, A549, Hep G2 and HeLa cells; in vivo: female BALB/c mice injected with 4T1 cells
[75]			EXOs from milk	Sequential centrifugations	Incubation	/	In vivo: athymic nude mice bearing subcutaneous lung cancer A549 xenografts
[76]			EXOs from milk	Sequential centrifugations	Sonication	Folic acid	In vitro: MCF-7 and MDA-MB-231 breast cancer cells
[77]			EXOs from human U87 glioblastoma cells	Exo-spin™ kit	Incubation/sonication	/	In vitro: U-87 glioblastoma cells
[78]	*Taxus brevifolia* (Taxaceae)	Paclitaxel	Hybrid NPs: poly(caprolactone) NPs covered with mouse red blood cell (RBC) membranes	Co-extrusion	The drug was dissolved with poly(caprolactone) during the NPs core preparation	/	In vitro: RAW 264.7 macrophages, 4T1 breast cancer cells; ex vivo: female BALB/c nude mice injected with 4T1 cells; in vivo: SD rats
[79]			Hybrid NPs: poly(caprolactone) NPs covered with mouse RBC membranes	Co-extrusion	The drug was dissolved with poly(caprolactone) during the NPs core preparation	DiR	In vitro: 4T1 breast cancer cells; ex vivo, in vivo: female BALB/c nude mice injected with 4T1 cells
[80]			Hybrid NPs: drug nanocrystals covered with mouse RBC membranes	Sonication	The core of the NPs is composed of nanocrystals of the active compound	/	In vitro: 293 T, HT29, HepG2, MDA-MB-486, MCF-7, 4T1 cancer cells; ex vivo, in vivo: female BALB/c nude mice injected with 4T1 cells
[81]			Hybrid NPs: carboxymethylcellulose and stearic acid core coated with mouse RBC membranes	Sonication	Incubation	Folic acid and PEG chains	In vitro: RAW 264.7 macrophages, HepG2, A549, and A375 cancer cells; ex vivo, in vivo: mouse models of lung cancer, liver cancer, and melanoma
[82]			Hybrid NPs: albumin NPs coated with RAW 264.7 murine macrophage membranes	Co-extrusion	The drug was dissolved in the solvent mixture during the NPs core preparation	/	In vitro: 4T1, A549, MCF-7, B16F10 cancer cells; ex vivo, in vivo: B16F10-bearing C57BL/6 mice
[83]			Hybrid NPs: Poly(lactic-co-glycolic acid) (PLGA) NPs coated with human HeLa cell membranes	Co-extrusion	The drug was dissolved with PLGA during the NPs core preparation	/	In vitro: HeLa, Ect1, LO2, and RAW 264.7 cells; ex vivo, in vivo: HeLa xenografted female BALB/c nude mice
[84]			Hybrid NPs: PLGA NPs coated with human osteosarcoma and mouse macrophage cell membranes	Sonication	The drug was dissolved with PLGA during the NPs core preparation	/	In vitro: 143B cells; ex vivo, in vivo: male BALB/c nude mice injected with 143B cells
[85]	*Camptotheca acuminata* (Nyssaceae)	Camptothecin	EXOs from human cervical cancer cells	Ultracentrifugation	Electroporation	/	In vitro: Ect1/E6E7, End1/E6E7, Vk2/E6E7 and HUCEC cervical epithelial cells; ex vivo, in vivo: BALB/c mice implanted with human cervical cancer tissue
[86]			Hybrid NPs: mouse RBC-derived droplets with a liquid core of perfluoro-n-pentane	Sequential centrifugations	The drug was mixed with RBC membranes and perfluoro-n-pentane during the droplet preparation	/	In vitro: primary mouse peritoneal macrophages, HeLa cells, BJAB human lymphoblastoid B cells; in vivo: male C57BL mice
[87]			Hybrid NPs: mouse RBC nanobubbles with a sulfur hexafluoride core	Centrifugation and sonication	Dubious	/	In vitro: C26 mouse colon carcinoma cells, CHO Chinese hamster ovary cells; in vivo: male BALB/c mice injected with C26 cells
[88]			Human RBC-derived vesicles	Extrusion	Incubation and extrusion	/	In vitro: adenocarcinomic human alveolar basal epithelial cells, A549 lung carcinoma cells and THP-1 human monocytes; in vivo: male BALB/c mice
[89]			Human RBC-derived vesicles	Extrusion	The drug was incubated with the photosensitizing agent before extrusion	ICG	In vitro: A549 cells; in vivo: female BALB/c mice subcutaneously inoculated with Ehrlich Ascites Carcinoma (EAC) cell suspension
[90]			Hybrid NPs: human RBC membrane-coated polymeric micelles	Extrusion	The drug was dissolved in the organic phase during the polymeric NPs core preparation	/	In vitro: A549 human adenocarcinomic alveolar basal epitheliel cells; in vivo: EAC mice
[91]			Hybrid NPs: mouse macrophage membrane-coated solid lipid NPs	Sequential centrifugations and extrusion	The prodrug was dissolved with the lipids during the lipid particles core formation	/	In vitro: B16F10 mouse melanoma and 4T1 mouse breast cancer cells; in vivo: BALB/c mice
[92]	*Curcuma longa* (Zingiberaceae)	Curcumin	EXOs from milk	Sequential centrifugations	Incubation	/	In vitro: H1299 and A549 human lung cancer cells, HeLa cells, MDA-MB-231 and T47D breast cancer cells; ex vivo: athymic nude mice injected with CaSki cervical cancer cells
[93]		Curcumin (and resveratrol)	EXOs from milk	Ultracentrifugation	Sonication/electroporation/ incubation	/	In vitro: MCF-7 human breast cancer cells; ex vivo: female Sprague–Dawley rats
[94]		Curcumin	EXOs from RAW 264.7 murine macrophages	Ultrafiltration and sequential centrifugations	Electroporation	NPR-1 glycoprotein	In vitro: U251 human malignant glioblastoma multiforme and Bel-7404 human hepatoma cells; in vivo: glioma-xenografted female BALB/c nude mice
[95]			EXOs from CT26 murine colon cancer cells and from erythrocytes	Ultracentrifugation	The drug was included in the mixture during the preparation of calcium carbonate NPs encapsulated in the exosomes	/	In vitro: CT26 mouse colon cancer cells, RAW 264.7 mouse macrophages; ex vivo, in vivo: BALB/c mice, CT26-injected BALB/c mice
[96]	*Catharanthus roseus* (Apocynaceae)	Vincristine	EVs from three types of human mesenchymal stem cells	Ultracentrifugation	Incubation	/	In vitro: U87MG human glioblastoma cells
[97]	Different sources: honey, propolis, *Passiflora caerulea* and *Passiflora incarnata* (Passifloraceae), *Oroxylum inolicum* (Bignoniaceae)	Chrysin	EVs from SCC9 human tongue squamous carcinoma cells	Sequential centrifugations	Priming of the parental cells with the drug	/	In vitro: SCC9 tongue squamous carcinoma cells; ex vivo, in vivo: female nude mice injected with SCC9 cells
[98]	Beans, berries, fruits, flowers (*Viola* and *Delphinium*)	Delphinidin	EVs from JAWS II murine dendritic cells	Sequential centrifugations	Incubation	/	In vitro: HAOEC human aortic endothelial cells
[99]	*Berberis vulgaris*, *Berberis aquifolium* and *Berberis aristate* (Berberidaceae)*, Tinospora cordifolia* (Menispermaceae)	Berberin	EVs from JAWS II murine dendritic cells	Sequential centrifugations	Impregnation	/	In vitro: MDA-MB-231 human breast cancer cells, HUVECs
[100]	Black beans extract	Soyasaponins, flavonoids	EXOs from 4 different human cancer cell lines	Total Exosome Isolation Reagent kit	Incubation/electroporation	/	In vitro: human hormone-dependent mammary (MCF7), prostate (PC3), colon (Caco2) and liver (HepG2) cancer cells
[101,102]	Bilberry extract	Anthocyanins	EXOs obtained from milk	Differential centrifugation	Incubation	/	In vitro: human lung (A549 and H1299), breast (MDA-MB-231 and MCF7), pancreatic (PANC1 and Mia PaCa2), prostate (PC3 and DU145), colon (HCT116) and ovarian (A2780, A2780/CP70, OVCA432 and OVCA433) cancer cells; in vivo: wild-type female C57BL/6 mice, female athymic nude mice

## Data Availability

Not applicable.
